# A systematic review and meta-analysis of food handling practices in Ghana vis-a-vis the associated factors among food handlers during 2009 and 2022

**DOI:** 10.1038/s41598-023-46150-8

**Published:** 2023-10-31

**Authors:** Lawrence Sena Tuglo, Snehasish Mishra, Ranjan K. Mohapatra, Nii Korley Kortei, John Nsor-Atindana, Henok Mulugeta, Qingyun Lu, Mavis Pearl Kwabla, Agabus Tetteh Patu, Tania Chaudhuri, Jessica Dzigbordi Tuglo, Subrata Narayan Das, Sylvia Mawusinu Sakre, Komla Sylvester Affram, Alfred Doku

**Affiliations:** 1 Department of Nutrition and Dietetics, School of Allied Health Sciences, University of Health and Allied Sciences, Ho, Ghana; 2 School of Biotechnology, Campus-11, KIIT Deemed-to-be University, Bhubaneswar, Odisha 751 024 India; 3 Department of Chemistry, Government College of Engineering, Keonjhar, Odisha 758 002 India; 4 School of Nursing and Midwifery, Faculty of Health, University of Technology Sydney, New South Wales, Australia; 5 Department of Epidemiology, School of Public Health, Nantong University, Nantong, Jiangsu China; 6 Department of Epidemiology and Biostatistics, Fred N. Binka School of Public Health, University of Health and Allied Sciences, Hohoe, Ghana; 7 Department of Animal Science, College of Agriculture and Natural Resource, Kwame Nkrumah University of Science and Technology, Kumasi, Ghana; 8 Department of Zoology, Dinabandhu Andrews College, University of Calcutta, Kolkata, West Bengal India; 9 Department of Midwifery, School of Nursing and Midwifery, University of Health and Allied Sciences, Ho, Ghana; 10 Department of Mining Engineering, Government College of Engineering, Keonjhar, Odisha 758 002 India; 11 Department of Community Health Nursing, Nurses Training College, Ho, Ghana; 12 Department of Internal Medicine, Ho Teaching Hospital, Ho, Ghana; 13 Department of Medicine and Therapeutics, University of Ghana Medical School, Accra, Ghana

**Keywords:** Cardiology, Diseases, Gastroenterology, Health care, Health occupations, Medical research, Risk factors

## Abstract

Foodborne diseases (FBDs) are a major public health concern, especially in Sub-Saharan African (SSA) countries, such as Ghana, where poor food handling practices (FHPs) are prevalent. To estimate the pooled proportion of good FHPs and the associated factors among Ghanaian food handlers, this systematic review and meta-analysis was conducted to aid scholars, practitioners and policymakers in devising FBD-preventable interventions. The scientific databases PubMed, Google Scholar, Science Direct, African Journals Online, ProQuest, and Directory of Open Access Journals were systematically searched until April 19, 2023, for relevant literature. Observational studies meeting the inclusion criteria of reported good FHPs among food handlers were included. Three authors independently searched the database, assessed the risks of bias and extracted the data from the shortlisted articles. A random-effects model with the DerSimonian and Laird model was used to estimate the pooled effect size of FHPs and the pooled odds ratio (POR) of FHP-associated factors. Out of the 2019 records collated, 33 with a total sample size of 6095 food handlers met the inclusion criteria for meta-analysis. The pooled proportion of good FHPs among Ghanaian food handlers was 55.8% [95% Cl (48.7, 62.8%); I^2^ = 97.4%; *p* < 0.001]. Lack of food safety training [POR = 0.10; 95% CI (0.03, 0.35); *p* = 0.001] and inadequate knowledge of food hygiene [POR = 0.36; 95% CI (0.01, 10.19); *p* < 0.001] were identified as the critical good FHP-associated factors. The study showed that the proportion of good FHPs among Ghanaian food handlers was 55.8%. To increase knowledge of food hygiene among food handlers, the Ghanaian Food and Drugs Authority (FDA) is recommended to provide regular training on food safety for the well-being of the general public.

## Introduction

In light of the various endemic infectious diseases, the health infrastructure and the associated food-handling practices in Ghana need critical consideration^1,2^. These include food processing and manufacturing facilities, storage and logistics services, as the majority of infectious diseases either emerge or spread through the consumption of unhygienic food with unacceptable levels of food safety hazards^3,4^. In the face of the prevailing low economic conditions and poor health infrastructure, it is prudent to practise all preventive measures through good food handling practices (FHPs) that could ensure safe food and drastically reduce the risk of foodborne diseases (FBDs)^5,6^.

FHPs are a collective key measure primarily to maintain biological food safety during storage, processing, preparation and the hygiene of cooking utensils as well as other surfaces that are likely to come in direct contact with food^7,8^. Poor FHPs across all economies are responsible for 75% of FBD outbreaks, resulting in approximately 420,000 deaths each year^6,9^. Research reveals that 18% of all FBD deaths were associated with food being contaminated by food handlers^6^. Food handlers are individuals who are directly in contact with food storage, preparation and packaging, as well as with food-handling utensils^8,10^.

Studies have shown that training in food safety^6,8,10^, food hygiene attitudes^8,10^, educational level^6,8,10^, knowledge of food hygiene^8^, average monthly income^6^ and registration of food handlers and their medical fitness^6,8,10^ are associated with good FHPs. Systematic reviews and meta-analyses conducted in Ethiopia revealed that the pooled good FHPs among food handlers ranged between 48.4^10^ and 50.5%^8^. The proportion of good FHPs among Ghanaian food handlers based on individual observational studies ranged between 24.4^19^ and 87.0%^2^. Frequent FBD outbreaks such as diarrhoea, cholera and typhoid in Ghana are presumably directly associated with poor FHPs and poor environmental sanitation^11–13^.

Researchers and policymakers need empirical evidence in decision-making. However, observational studies of individual research groups on the estimated proportion of FHPs in Ghana are inconsistent for an informed decision. Given these gaps, it was necessary to undertake a systematic review and meta-analysis of FHPs in Ghana to adequately evaluate them. This review considers good FHPs to be the reported standard FHPs classified as ‘good’ based on statistical analyses. Good FHP ensures that the food for consumption is largely safe from biological hazards^2,4,6^.

A meta-analysis was therefore conducted to pool the proportion of good FHPs among Ghanaian food handlers to generate a single summary estimate from several independent studies by pooling the data. It increases the sample size, detects publication biases and leads to more precise estimates of the proportion while identifying deficiencies in study design, data analyses and interpretation of the findings. Estimating the pooled proportion of good FHPs and the associated factors among Ghanaian food handlers was the primary objective to aid scholars, practitioners and policymakers in devising FBD-preventable interventions.

The findings could also help health authorities and agencies such as the Food and Drugs Authority (FDA), especially in Ghana, to implement good FHPs among food handlers to prevent FBDs. Furthermore, this review could be beneficial for international agencies such as the World Health Organization (WHO) and the Food and Agriculture Organization (FAO) of the United Nations (UN) to develop an effective global food safety plan.

## Methods

This systematic review and meta-analysis report followed the Preferred Reporting Items for Systematic Reviews and Meta-Analyses (PRISMA 2020) guidelines^14^. The protocol that was followed is registered on PROSPERO (ID: CRD42022352777).

### Search strategy

Relevant literature was searched online on PubMed, Google Scholar, Science Direct, African Journals Online, ProQuest, and the Directory of Open Access Journals for the available published articles until April 19, 2023, using ‘food’, ‘foods’, ‘handling practice’, ‘hygiene practice’, ‘hand hygiene’, ‘safety practice’, ‘food hygiene’, ‘food handling’, ‘food safety’, ‘food sanitation’, ‘professional practices’, ‘associated factors’, ‘identified factors’, ‘factors associated’, ‘determinant factors’, ‘factors contributing’, ‘food handlers’, ‘food vendors’, ‘street food vendors’ and ‘Ghana’ as keywords and Medical Subject Headings (MeSH). A complete list of the search keywords and the strategies adopted are detailed in the supplementary file (Tables 1, 2). In addition to the database search, the cited literature listed in the reference of the articles was also manually searched, and the relevant additional articles were identified and included.

**Table 1:** Characteristic patterns of all the included literature in the study.

Region	Study design	Sampling method	Sample size	Response rate	Proportion of good FHPs	Risk of bias	References
Ashanti	CS	SR	60	100	53.4	Moderate	Monney et al.^42^
Ashanti	CS	SR	81	100	54.6	Low	Gyebi et al.^22^
Ashanti	CS	Convenience	125	100	54.1	Moderate	Dwumfour-Asare^29^
Ashanti	CS	SR	340	100	85.2	Moderate	Addo-Tham et al.^7^
Bono East	CS	SR	100	100	40	Moderate	Dajaan et al.^36^
Brong Ahafo	CS	Purposive	140	100	24.4	Moderate	Amaami et al.^31^
Central	CS	Systematic	306	100	68.2	Low	Odonkor et al.^4^
Eastern	CS	SR	30	100	61.8	Moderate	Antwi^19^
Eastern	CS	Purposive	40	100	77.5	Moderate	Nartey et al.^24^
Greater Accra	CS	Systematic	127	50.8	35.4	Low	Donkor et al.^20^
Greater Accra	CS	SR	104	96.2	41.8	Moderate	Oduro-Yeboah et al.^2^
Greater Accra	CS	Systematic	278	100	52	Moderate	Kunadu et al.^27^
Greater Accra	CS	SR	132	86.8	33	Moderate	Ovai et al.^28^
Greater Accra	CS	Convenience	50	100	60.5	Moderate	Odonkor et al.^35^
Greater Accra	CS	Systematic	200	100	64	Moderate	McArthur-Floyd et al.^38^
Northern	CS	Convenience	206	68.2	66	Low	Amegah et al.^40^
Northern	CS	SR	150	100	49	Moderate	Danikuu et al.^41^
Northern	CS	SR	100	100	52.1	Moderate	Adzitey et al.^21^
Northern	CS	SR	199	99.5	65.3	Moderate	Ziblim et al.^12^
Northern	CS	SR	200	100	86.9	Low	Apanga et al.^34^
Upper West	CS	Systematic	266	30.9	87	Moderate	Dun-Dery et al.^39^
Upper West	CS	Purposive	30	100	68.5	Moderate	Mwini et al.^17^
Volta	CS	SR	407	96.2	62.9	Low	Tuglo et al.^6^
Volta	CS	SR	65	100	72.1	Moderate	Dah^23^
Volta	CS	SR	97	100	48.8	Moderate	Appietu et al.^25^
Volta	CS	Convenience	608	100	51.6	Low	Madilo et al.^11^
Volta	CS	SR	275	100	83.6	Moderate	Frempong et al.^30^
Volta	CS	SR	97	100	25.3	Moderate	Bormann et al.^32^
Western	CS	Purposive	50	100	29.6	Moderate	Boakye et al.^18^
Two or more	CS	SR	720	100	32.6	Moderate	Bigson et al.^16^
Two or more	CS	Systematic	235	100	46.6	Low	Akabanda et al.^26^
Two or more	CS	SR	200	100	66.5	Moderate	Monney et al.^33^
Two or more	CS	Purposive	77	100	37.5	Moderate	Annan-Prah et al.^37^

CS, cross-sectional; SR, simple random.

**Table 2:** Subgroup analyses of the proportion of good FHPs among Ghanaian food handlers.

Subgroup analysed	No. of the study included	Good FHP proportion [95% CI]	Heterogeneity across studies		Heterogeneity among groups (*p* value)
			I^2^ (%)	*p* Value	
Overall	33	55.75 [48.65, 62.84]	97.39	< 0.001	< 0.001
Year of publication					0.430
< 2020	21	53.53 [43.79, 63.27]	96.88	< 0.001	
≥ 2020	12	59.53 [48.25, 70.82]	98.12	< 0.001	
Sampling method					0.445
Nonprobability	9	51.78 [41.01, 62.54]	92.99	< 0.001	
Probability	24	57.13 [48.58, 65.68]	97.79	< 0.001	
Sample size					0.627
> 300	5	60.08 [40.83, 79.34]	99.09	< 0.001	
≤ 300	28	54.94 [47.17, 62.71]	96.51	< 0.001	
Risk of bias					0.525
Low	8	59.23 [48.40, 70.05]	96.60	< 0.001	
Moderate	25	54.63 [45.43, 63.82]	97.64	< 0.001	

### Eligibility criteria

Random studies on food handlers operating in food catering establishments, institutions and roadside/streets were included. Observational studies on cross-sectional, case–control and cohort studies that reported the proportion of good FHPs (or provided data on good FHPs of food handlers for which the proportion could be calculated as the primary outcome) were included. The shortlisted articles included those published across the timeline in the English language and excluded inaccessible full-text articles where several attempts to communicate with the corresponding authors failed. It also excludes articles where it was difficult to extract the needed data of the primary objective, i.e., the proportion of good FHPs. Additionally, studies outside Ghana and articles with ambiguous methodologies were also excluded.

### Quality assessment of the shortlisted studies

Three authors independently assessed the quality of the studies and resolved the discrepancies that arose through consensus. The Joanna Briggs Institute (JBI) quality assessment tool for prevalence studies was adopted to assess the quality of the shortlisted studies/data and the risks of bias^15^. The JBI tool was chosen because it helped assess the methodological quality of a study and determine the extent to which a study has addressed the possibility of bias in its design, conduct and analysis^15^. It also aided in reducing information overload by eliminating irrelevant and/or weak studies and allowed the identification of the most relevant work^15^. The tool comprises nine parameters focusing on the appropriate sampling frame, proper sampling technique, adequate sample size, study subject and setting description, sufficient data analysis, use of valid methods to identify conditions, validation of all participants, use of appropriate statistical analysis and adequate response rate. The risks of bias were classified based on the total score; a score of 0 was assigned if the parameters coincided and 1 if they did not. The risk was low with a score of ≤ 2, moderate at 3–4, or high at ≥ 5. Only the articles with low and moderate risks of bias were included, as detailed in Table 3 in the supplementary file. Disagreements, if any, were resolved through discussion and consensus.

**Table 3:** Meta-regression analyses to assess the causes of heterogeneity of the findings in the included literature.

Variable	Bivariate			Multivariate		
	Coefficient	[95% CI]	*p* Value	Coefficient	[95% CI]	*p* Value
Year of publication	0.848	[− 1.41, 3.11]	0.462	1.135	[− 1.53, 3.80]	0.404
Sample size	0.008	[− 0.04, 0.05]	0.727	− 0.004	[− 0.06, 0.05]	0.891
Response rate	− 0.162	[− 0.62, 0.30]	0.490	− 0.215	[− 0.72, 0.29]	0.402
Risks of bias score	− 0.948	[− 7.73, 5.84]	0.784	− 0.441	[− 7.66, 6.78]	0.905

### Screening, selection, and data extraction from the shortlisted studies

Mendeley desktop Ver. 1.19.6 was used to import all the references from the searched database. Three authors used Rayyan software to screen the title, abstract, full text and study selection. A standard extraction format was used to extract the necessary data, such as first author, publication year, region, study design, sampling method, sample size, response rate and good FHP proportions (the standard FHPs reported in the individual studies classified as good based on the categorization of the statistical analyses were considered good FHPs). For instance, hygiene practices of 37.1% were classified as ‘poor’ and 62.9% as ‘good’ by Tuglo et al.^6^. ‘Good hygiene practices’ were extracted from all the included literature. Any screening, study selection, and data extraction disagreements were resolved through consensus.

### Statistical analyses

Data were analysed using STATA software version 17. The heterogeneity of the dataset was assessed using Cochran’s Q test and I^2^ statistic with the corresponding *p* values. The random-effects model with the DerSimonian and Laird (DL) model was used to estimate the pooled effect size of FHPs and the pooled odds ratio (POR) of the FHP-associated factors. A leave-one-out sensitivity analysis was conducted to assess the strength and influence of each study on the overall effect size estimate. A funnel plot was used visually to assess publication bias in conjunction with statistical methods such as the regression-based Egger test and Begg’s rank correlation tests (*p* < 0.05) for confirmation. Subgroup analysis and meta-regression were conducted to identify potential sources of heterogeneity in the pooled proportion estimates.

## Results

**Figure 1:**
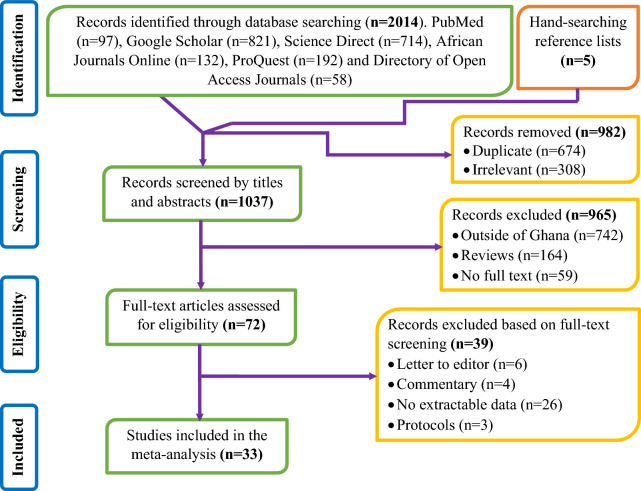
PRISMA flow diagram specifying the considerations to exclude and include the articles.

Database searches of the relevant studies yielded 2014 records, and manual searching from the lists of references yielded an additional five records. A total of 1037 titles and abstracts were screened after removing 982 duplicate and/or irrelevant records and excluding an additional 965 records without full texts, and 72 full-text records were considered for data assessment. Of these, 39 articles were finally excluded where incoherence was discovered during data assessment, and the data of 33 studies were meta-analysed (Fig. 1).

### Characteristics of the shortlisted studies

The 33 studies^2,4,6,7,11,12,16–42^ included were published between 2009 and 2022 and were cross-sectionally designed with a total of 6095 food handlers. Six major studies were carried out in the Greater Accra^2,20,27,28,35,38^ and Volta^6,11,23,25,30,32^ regions, and five were carried out in the Northern region^12,21,34,40,41^. Eighteen studies^2,6,7,12,16,19,21–23,25,28,30,32–34,36,41,42^ used a simple random sampling technique, and^4,20,26,27,38,39^ used systematic sampling (18.2%; n = 6) (Table 1).

### Risk of bias in the included studies

Regarding the quality of the included studies, eight studies^4,6,11,20,22,26,34,40^ had a low risk of bias (24.2%), and 25 studies^2,7,12,16–19,21,23–25,27–33,35–39,41,42^ had a moderate risk of bias (75.8%) (Supplementary File Table 3, on pages 2 and 3).

### Meta-analysis

**Figure 2:**
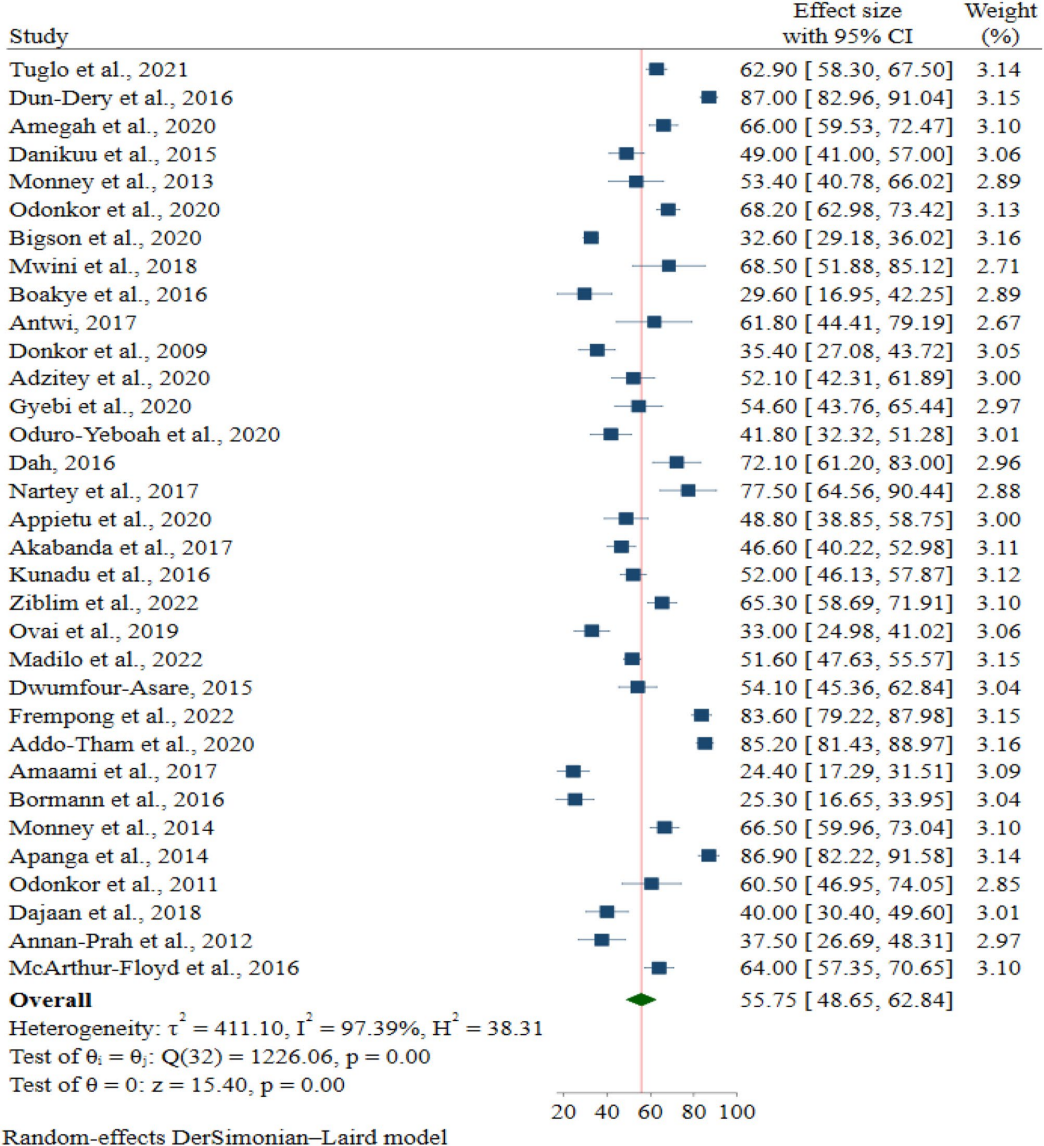
Forest plot of the proportion of good FHPs among food handlers in Ghana.

The pooled proportion of good FHPs from the meta-analysis on Ghanaian food handlers was 55.8% (95% CI 48.7, 62.8%). The heterogeneity across studies was high and significant [(I^2^ = 97.4%); *p* < 0.001)]. Based on the included studies, the highest proportion of good FHPs was 87.0% (95% CI 83.0, 91.0%), as reported by Dun-Dery et al.^2^, and the lowest was 24.4% (95% CI 17.3, 31.5%), as reported by Amaami et al.^19^ (Fig. 2).

### Sensitivity analysis

**Figure 3:**
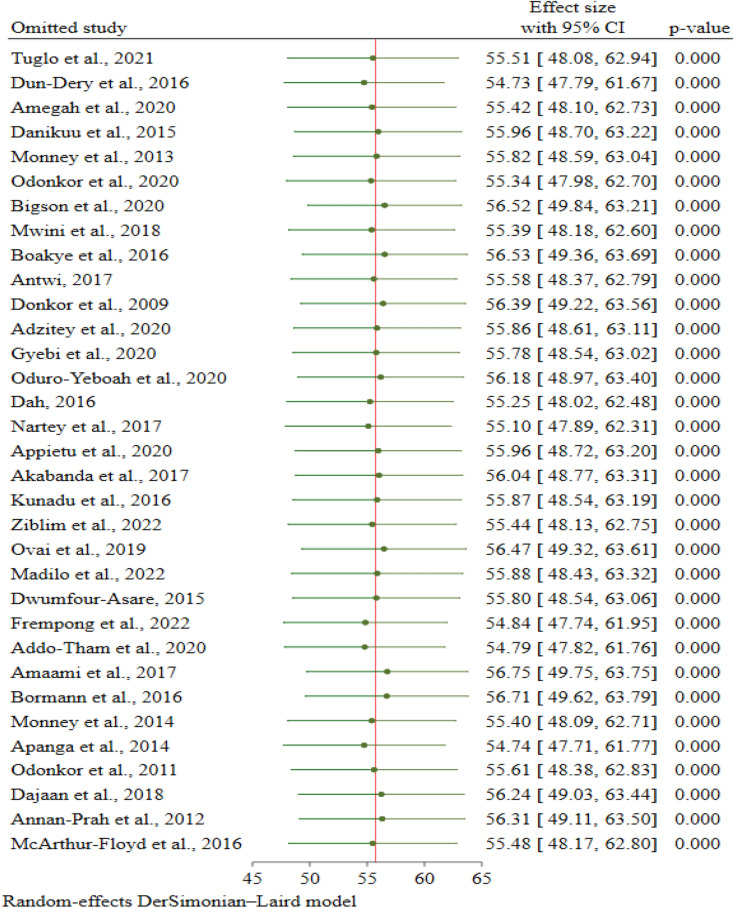
Sensitivity analysis of the proportion of good FHPs among food handlers in Ghana.

To estimate the influence of individual studies on the overall meta-analysis, a sensitivity analysis was performed. The pooled data meta-analysis results were close to the actual effect size, which ranged from 54.7% (95% CI 47.8, 61.7%; *p* < 0.001) to 56.8% (95% CI 49.8, 63.8%; *p* < 0.001), even after the removal of a single study at a time, suggesting that no single included study had an overwhelming effect on the pooled estimate of good FHPs (Fig. 3).

### Subgroup analysis

The proportion of good FHPs in studies reported in or after 2020 was 59.5% (95% CI 48.3, 70.8%), and the same was 53.5% (95% CI 43.8, 63.3%) in studies reported earlier. The proportion of good FHPs was 57.1% (95% Cl 48.6, 65.7%) in studies using probability sampling, while it was 51.8% (95% CI 41.0, 62.5%) in studies that did not. The proportion of good FHPs was 60.1% (95% CI 40.8, 79.3%) among studies having > 300 and 54.9% (95% CI 47.2, 62.7%) among studies having ≤ 300 sample sizes. Studies assessed as having low risks of bias showed a high (59.2%) proportion of good FHPs (95% CI 48.4, 70.1%) compared with moderate (54.6%) risks of bias (95% CI 45.4, 63.8%). All the subgroups had substantial heterogeneity. The heterogeneity of good FHP estimates for each subgroup could not be explained, as the results in individual articles were inconsistent (Table 2).

### Meta-regression

Meta-regression was executed using variables such as the year of publication, sample size, response rate and risk of bias score to identify potential sources of heterogeneity. Bivariate and multivariable analyses showed insignificant sources of heterogeneity among them (*p* > 0.05; Table 3).

### Publication bias

**Figure 4:**
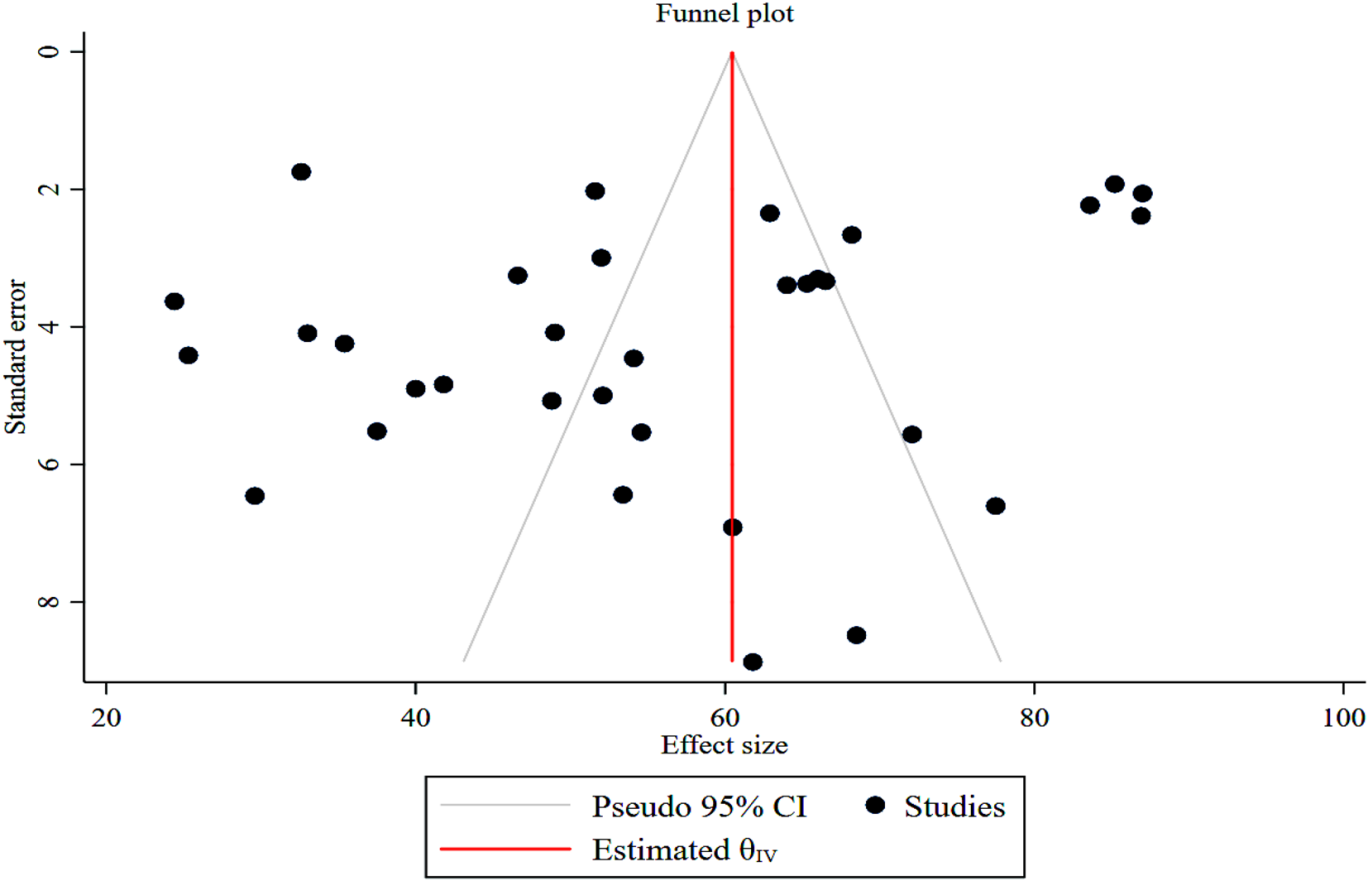
Funnel plot of studies with risk bias of the proportion of good FHPs among Ghanaian food handlers.

The visible symmetric funnel shape plot suggested that there was no publication bias (Fig. 4). The regression-based Egger test (*p* = 0.378) and Begg’s rank correlation test (*p* = 0.486) meta-analyses confirmed that there was no publication bias among the included studies.

### Factors associated with FHPs among Ghanaian food handlers

**Figure 5:**
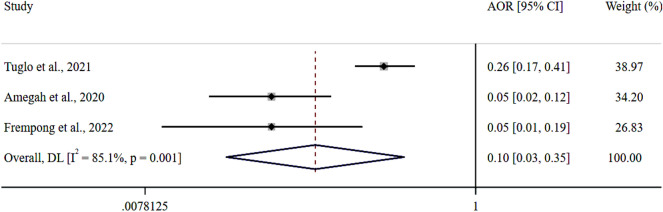
Forest plot of the association between lack of food safety training and good FHPs in Ghana.

**Figure 6:**
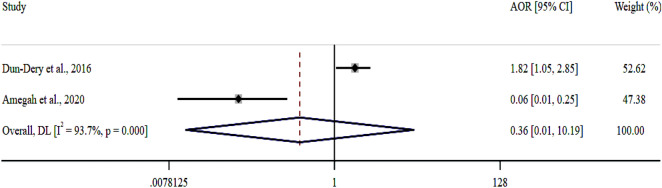
Forest plot of the association between inadequate knowledge of food hygiene and good FHPs in Ghana.

Four out of 33 included studies^6,30,39,40^ reported FHP-associated factors (Table 4). Three studies^6,30,40^ reported an association between a lack of food safety training and good FHPs. The pooled estimate indicated that the odds of abiding by good FHPs were 0.10× lower among the nontrained food handlers than among their trained counterparts (POR = 0.10; 95% CI 0.03, 0.35; *p* = 0.001; Fig. 5). Two studies^39,40^ reported that inadequate knowledge of food hygiene and good FHPs were associated. The POR showed that food handlers with inadequate knowledge of food hygiene were 0.36× less likely to adhere to good FHPs than those with adequate knowledge (POR = 0.36; 95% CI 0.01, 10.19%; *p* < 0.001; Fig. 6).

**Table 4:** Factors associated with FHPs among Ghanaian food handlers.

Number	Factors associated with FHPs	Study	Odds ratio [95% Cl]
1	Lack of food safety training	Tuglo et al.^6^	0.26 [0.17, 0.41]
		Amegah et al.^40^	0.05 [0.02, 0.12]
		Frempong et al.^30^	0.05 [0.01, 0.19]
2	Inadequate knowledge of food hygiene	Dun-Dery et al.^39^	1.82 [1.05, 2.85]
		Amegah et al.^40^	0.06 [0.01, 0.25]
3	Registered as food verdor	Tuglo et al.^6^	7.50 [4.27, 13.19]
4	Average monthly income	Tuglo et al.^6^	4.89 [1.59, 15.34]
5	Secondary level of education	Tuglo et al.^6^	4.06 [1.63, 10.11]

## Discussion

The majority of FBDs are often associated with poor FHPs^6^. This meta-analysis showed 55.8% good FHPs among the Ghanaian food handlers, implying that the recommended personnel hygiene, utensils and surroundings cleaning and appropriate food storage being crucial in food poisoning and FBDs reduction were complied with. This finding could be attributed to adequate food hygiene training, proper infrastructure and adequate supervision by regulatory agencies such as the FDA. Educational campaigns and action-oriented interventions before and after food safety training by the FDA and monitoring the effects could improve FHPs and ensure food safety at the consumer level.

Consistent with our findings, an earlier meta-analysis (50.5%) by Zenbaba et al.^8^ was reported among Ethiopian food handlers. The pooled estimate in the present study was higher than the 48.4% figure of Negassa et al.^10^, who carried out a pooled good FHP meta-analysis in Ethiopia. The disparities across the studies are attributable to environmental and cultural differences, access to food safety training, varying knowledge of food hygiene and the frequency of supervision by the enforcement agencies. A systematic review reported 72.7% poor FHPs among Bangladeshi food handlers^43^, wherein the discrepancy could be due to the diverse study setting and sociodemographic characteristics among the food handlers. Food establishments operate without formal food safety training, registration to operate food business or regular medical check-ups and fitness tests of the food handlers in most developing countries^6,30,40^.

The heterogeneity among the included literature was significant, as reflected through analyses of the subgroup ‘year of publication’ and the sampling method. The significant statistical heterogeneity arising from the methodological differences in subgroup analyses suggested that all the studies did not estimate the same quantity but does not necessarily suggest that the effect of the pooled estimate size varied. A high proportion of good FHPs was seen in studies published after 2020. This anomaly is attributable to the individual good FHP proportions included in the meta-analysis, sampling method and differences in the study setting. Another reason could be that FBD prevention measures during the COVID-19 pandemic, as enforced by the WHO, warranted adherence to personnel hygiene^44^ in line with the WHO’s five keys to safe food^5^. The meta-analysis in the subgroup ‘probability sampling’ had a high proportion of good FHPs compared to its nonprobability counterpart. This disparity is attributed to the varying sociodemographic characteristics of food handlers and the sampling techniques adopted.

**Figure 7:**
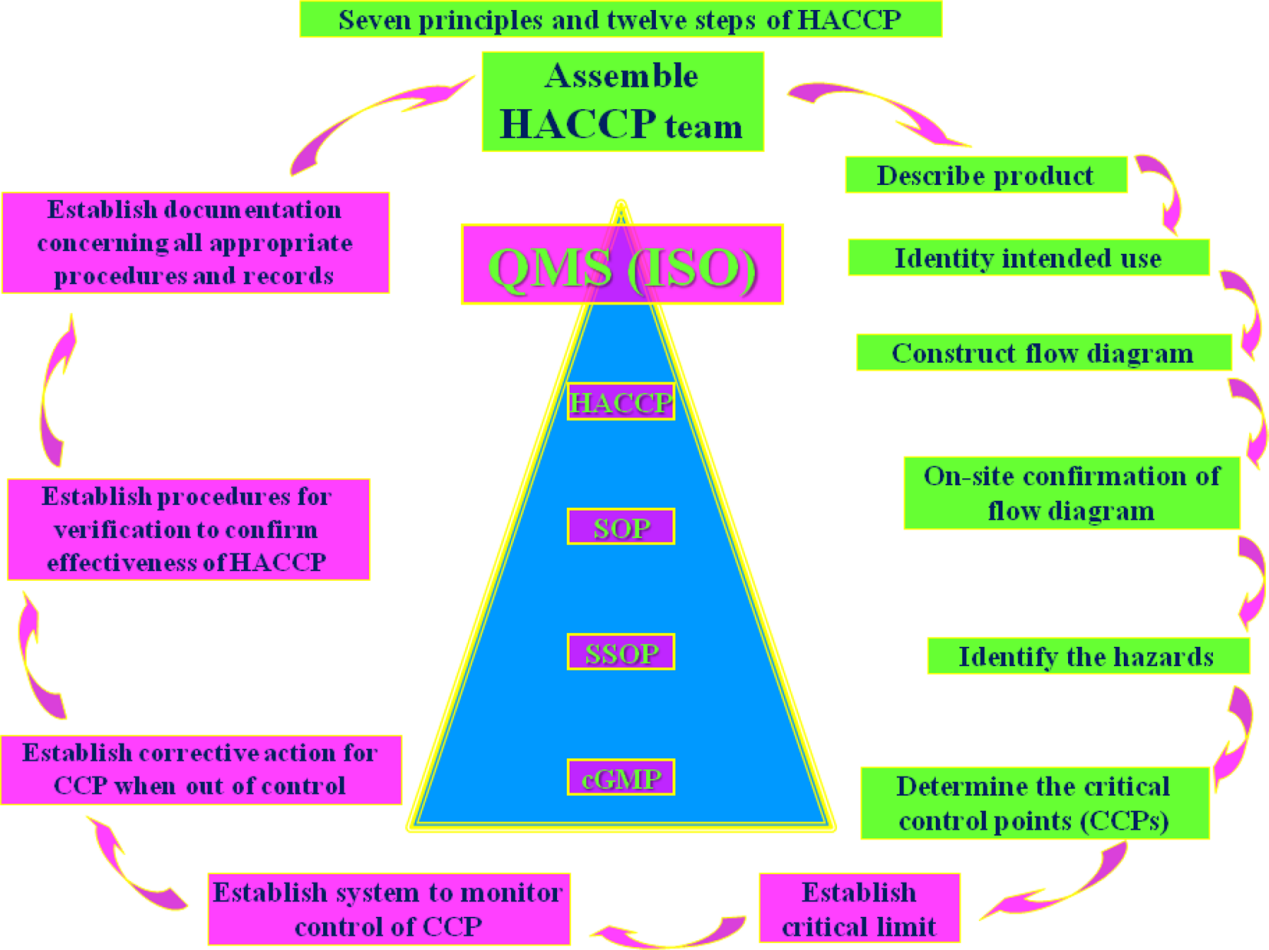
The quality assurance pyramid that defines and ensures the highest order of food safety.

A training of food handlers usually seeks to impart two major transformations, to acquire adequate knowledge and skillsets and to help translate the knowledge into practice^6^. The pooled odds ratio showed that food handlers with no food safety training were less likely to follow good FHPs than those who underwent them. This finding is corroborated by two earlier FHP meta-analyses conducted among Ethiopian food handlers^8,10^, which concluded that trained food handlers were more likely to follow good hygiene practices than untrained food handlers^8,10^. The training helped them gain accurate knowledge of good FHPs. Therefore, food handlers must receive frequent and effective training in food safety to ensure good FHPs under the cGMP (current Good Manufacturing Practices) requirements (Fig. 7).

As per the present meta-analysis, food handlers with inadequate knowledge of food hygiene were less likely to adhere to good FHPs than those with adequate knowledge. This concurs with an earlier Ethiopian meta-analysis that found that food handlers with good food hygiene knowledge were more likely to apply good FHPs than their ignorant counterparts^8^. Poor FHPs could critically affect the health of food consumers, with severe food poisoning and consequently the spread of FBDs; hence, our finding affirms the recommendation by the WHO^11^ of frequent assessment of FHP knowledge to prevent FBDs.

### Strengths and limitations

This is the first systematic review and meta-analysis on food safety and hygiene practices in the Ghanaian context to provide a pooled estimate of good FHPs and the associated factors to aid in the implementation of feasible FHP compliance and interventions among food handlers. Like any other scientific investigation, it has its limitations. First, the study was cross-sectional in design without permitting discrepancies between the cause and the effect. Second, the good FHP proportions extracted from the shortlisted literature were based on the reporting as presented, which might have a social desirability bias. Third, there were variations among the studies without a standard definition for ‘good FHPs’. Fourth, only articles published in the English language were included, excluding vernacular language literature.

### Summary of the finding

Any good food processing or manufacturing facility in modern times should ideally have cGMP and Hazard Analysis Critical Control Point (HACCP) in place in compliance with the food safety standards, the former occupying the base and the latter occupying the peak of the food safety (quality assurance) pyramid. The former focuses primarily on the training to be provided to the food handlers, and the latter focuses on analysing the possible physical, chemical or biological hazards that are likely in the food being processed (Fig. 7).

## Conclusions

The study showed that the proportion of good FHPs among Ghanaian food handlers was 55.8%. Lack of food safety training and inadequate knowledge of food hygiene were identified as good FHP-associated factors. To increase knowledge of food hygiene among food handlers, the FDA in Ghana is recommended to provide regular training on food safety for the well-being of the general public. The FDA should also be strict on food safety regulations among food handlers through surveillance and frequent monitoring systems to prevent frequent outbreaks of FBDs such as diarrhoea, cholera and typhoid in Ghana. Further studies in Ghana should focus on strong study designs such as cohort and interventional studies in reporting FHPs and should associate the adverse findings, if any, with region-specific FBD outbreaks.

### Supplementary Information

Supplementary Information.

## Data Availability

The manuscript contains all pertinent information.

## Abbreviations

FBDs: Foodborne diseases
SSA: Sub-Saharan African
FHPs: Food handling practices
POR: Pooled odds ratio
DL: DerSimonian‒Laird
CI: Confidence interval
FDA: Food and Drugs Authority
WHO: World Health Organization
COVID-19: Coronavirus disease 2019
cGMP: Current good manufacturing practice
SSOP: Sanitation standard operating procedure
SOP: Standard operating procedure
HACCP: Hazard analysis critical control point
QMS: Quality management system
ISO: International Organization for Standardization
