# Supplementary material for: A systematic review and meta-analysis of food handling practices in Ghana vis-a-vis the associated factors among food handlers during 2009 and 2022
Source: Sci Rep. 2023 Oct 31;13:18748. doi: 10.1038/s41598-023-46150-8 (PMC10618560; doi:10.1038/s41598-023-46150-8)
Supplement: Supplementary file 1 — Supplementary Information. [file 41598_2023_46150_MOESM1_ESM.doc]

Supplementary Table 1 Search keywords

| Keyword | Synonyms |
| --- | --- |
| Food | “food” OR “foods” |
| Handling practices | “handling practice” OR “hygiene practice” OR “hand hygiene” OR “safety practice” OR “food hygiene” OR “food handling” OR “food safety” OR “food sanitation” OR “professional practices” |
| Associated factors | “associated factors” OR “identified factors” OR “factors associated” OR “determinant factors” OR “factors contributing” |
| Food handlers | “food handlers” OR “food vendors” OR "street food vendors" |
| Ghana | “Ghana” |

Supplementary Table 2 Search strategies

| No | Database search | Search strategy | Search results |
| --- | --- | --- | --- |
| 1 | PubMed | ((("food"[MeSH Terms] OR food*[tiab]) AND ((("Hand Hygiene"[Mesh]) OR ("Food Safety"[Mesh])) OR ("Professional Practice"[Mesh]))) AND (("factors associated"[All Fields]) OR (determinant[All Fields] OR factors[All Fields]))) AND ("Ghana"[Mesh]) | 97 |
| 2 | Google Scholar | "food" OR “foods” AND “handling practice” OR “hygiene practice” OR "hand hygiene" OR “food handling” OR "food safety" OR “food sanitation” OR "professional practice" AND "factors associated" OR "determinant factors" OR “identified factors” AND “food handlers” OR "street food vendors" OR “food vendors” AND "Ghana" | 821 |
| 3 | Science Direct | Find articles with the keywords: "food" AND "food handlers" AND "handling practice" OR "hygiene practice" AND "factors associated" AND "Ghana" | 714 |
| 4 | African Journals Online | Text –word search: "food" AND "hygiene practice" OR "food hygiene" OR "handling practice" AND "Ghana” | 132 |
| 5 | ProQuest | Text –word search: "food handlers" AND "hygiene practice" AND "Ghana" | 192 |
| 6 | Directory of Open Access Journals | Find articles with the keywords: "food" AND "hygiene practice" OR “food hygiene" AND "Ghana” | 58 |
|  | Total |  | 2014 |

Supplementary Table 3 Quality assessment of the 33 included studies published between 2009 and 2022

| **No** | **Study** | **Sampling frame** | **Sampling strategy** | **Sample size** | **Description of research setting & population** | **Data analysis conducted with sufficient coverage** | **Valid methods used for the identification of the condition** | **Reliability of the instrument used** | **Statistical analysis methods** | **Response rate** | **Total** | **Risk of bias** |
| --- | --- | --- | --- | --- | --- | --- | --- | --- | --- | --- | --- | --- |
| 1 | Tuglo et al., 2021 | 0 | 0 | 0 | 0 | 0 | 0 | 0 | 0 | 0 | 0 | Low |
| 2 | Dun-Dery et al., 2016 | 1 | 0 | 0 | 0 | 0 | 1 | 1 | 1 | 0 | 4 | Moderate |
| 3 | Amegah et al., 2020 | 0 | 0 | 0 | 0 | 0 | 1 | 1 | 0 | 0 | 2 | Low |
| 4 | Danikuu et al., 2015 | 0 | 0 | 1 | 0 | 1 | 1 | 1 | 0 | 0 | 4 | Moderate |
| 5 | Monney et al., 2013 | 0 | 1 | 0 | 1 | 0 | 1 | 1 | 0 | 0 | 4 | Moderate |
| 6 | Odonkor et al., 2020 | 0 | 0 | 0 | 0 | 0 | 1 | 1 | 0 | 0 | 2 | Low |
| 7 | Bigson et al., 2020 | 1 | 1 | 0 | 0 | 0 | 1 | 1 | 0 | 0 | 4 | Moderate |
| 8 | Mwini et al., 2018 | 0 | 1 | 0 | 1 | 0 | 0 | 1 | 0 | 0 | 3 | Moderate |
| 9 | Boakye et al., 2016 | 0 | 0 | 1 | 0 | 0 | 1 | 1 | 0 | 0 | 3 | Moderate |
| 10 | Antwi, 2017 | 0 | 1 | 1 | 0 | 0 | 1 | 1 | 0 | 0 | 4 | Moderate |
| 11 | Donkor et al., 2009 | 0 | 0 | 0 | 0 | 0 | 1 | 1 | 0 | 0 | 2 | Low |
| 12 | Adzitey et al., 2020 | 0 | 0 | 0 | 1 | 0 | 1 | 1 | 0 | 0 | 3 | Moderate |
| 13 | Gyebi et al., 2020 | 0 | 0 | 0 | 0 | 0 | 1 | 1 | 0 | 0 | 2 | Low |
| 14 | Oduro-Yeboah et al., 2020 | 0 | 0 | 1 | 0 | 0 | 1 | 1 | 0 | 0 | 3 | Moderate |
| 15 | Dah, 2016 | 0 | 0 | 1 | 0 | 1 | 0 | 1 | 0 | 0 | 3 | Moderate |
| 16 | Nartey et al., 2017 | 1 | 0 | 0 | 0 | 0 | 1 | 1 | 1 | 0 | 4 | Moderate |
| 17 | Appietu et al., 2020 | 0 | 0 | 1 | 0 | 1 | 1 | 1 | 0 | 0 | 4 | Moderate |
| 18 | Akabanda et al., 2017 | 0 | 0 | 1 | 0 | 0 | 0 | 1 | 0 | 0 | 2 | Low |
| 19 | Kunadu et al., 2016 | 0 | 1 | 0 | 1 | 0 | 1 | 1 | 0 | 0 | 4 | Moderate |
| 20 | Ziblim et al., 2022 | 0 | 0 | 0 | 0 | 0 | 1 | 1 | 1 | 0 | 3 | Moderate |
| 21 | Ovai et al., 2019 | 1 | 1 | 0 | 0 | 0 | 1 | 1 | 0 | 0 | 4 | Moderate |
| 22 | Madilo et al., 2022 | 0 | 0 | 0 | 0 | 0 | 0 | 0 | 0 | 0 | 0 | Low |
| 23 | Dwumfour-Asare, 2015 | 0 | 0 | 1 | 0 | 0 | 1 | 1 | 0 | 0 | 3 | Moderate |
| 24 | Frempong et al., 2022 | 0 | 1 | 1 | 0 | 0 | 1 | 1 | 0 | 0 | 4 | Moderate |
| 25 | Addo-Tham et al., 2020 | 0 | 1 | 0 | 1 | 0 | 1 | 1 | 0 | 0 | 4 | Moderate |
| 26 | Amaami et al., 2017 | 1 | 0 | 1 | 0 | 0 | 1 | 1 | 0 | 0 | 4 | Moderate |
| 27 | Bormann et al., 2016 | 0 | 0 | 1 | 1 | 0 | 1 | 1 | 0 | 0 | 4 | Moderate |
| 28 | Monney et al., 2014 | 0 | 0 | 1 | 0 | 0 | 1 | 1 | 0 | 0 | 3 | Moderate |
| 29 | Apanga et al., 2014 | 0 | 0 | 0 | 0 | 0 | 1 | 1 | 0 | 0 | 2 | Low |
| 30 | Odonkor et al., 2011 | 0 | 0 | 1 | 0 | 0 | 1 | 1 | 0 | 0 | 3 | Moderate |
| 31 | Dajaan et al., 2018 | 0 | 0 | 0 | 1 | 0 | 1 | 0 | 1 | 0 | 3 | Moderate |
| 32 | Annan-Prah et al., 2012 | 0 | 1 | 0 | 0 | 0 | 1 | 1 | 0 | 0 | 3 | Moderate |
| 33 | McArthur-Floyd et al., 2016 | 0 | 0 | 1 | 1 | 0 | 1 | 1 | 0 | 0 | 4 | Moderate |
